# Life resists chemistry not conditions

**DOI:** 10.1093/femsmc/xtag041

**Published:** 2026-07-23

**Authors:** Adrienne Kish

**Affiliations:** Unit Communication Molecules and Adaptation of Microorganisms, Muséum National d’Histoire Naturelle (MNHN), CNRS; CP 54, 57 rue Cuvier, 75005 Paris, France

## Abstract

Extremophiles are used to study both the limits of life and its potential elsewhere. Categories are commonly employed to describe extreme conditions, however, changing the focus to the underlying chemical alterations of cellular biomolecules permits a clearer delineation of the limits of life. This also provides a common basis for interdisciplinary studies of life in extreme environments intersecting biology, chemistry, geology, environmental and planetary sciences. Using this perspective, four general principles can be drawn from the past decades of research into extremophiles: 1-Microbial survival after exposure to environmental stressors is challenged by damage incurred at the molecular level, 2-Damage-susceptibility of cellular (macro)molecules vary by structure, chemical composition, and surrounding molecules, 3-Local physicochemical states alter the chemistry underpinning microbial survival, and 4-The same macromolecular chemical and/or structural damages produced by different stressors can be resisted by the same cellular mechanisms. These general principles provide a common basis for interdisciplinary studies of the limits of life. This chemistry-focused approach has implications for defining the limits of life, paleoenvironment reconstruction, site selection for sampling, and choice of appropriate model systems for development of detection methods for microbial cells and extraction of molecules. This approach also contributes to the field of astrobiology, constraining the concept of habitability in the solar system and exoplanets. Descriptive terms are useful in science, but for extremophile microbiology and astrobiology, it is useful to recognize that life resists chemistry, not conditions.

## Teaser

Microbial survival is based on the nature and extent of damage to key cellular macromolecules incurred by exposure to different stressors, rather than the environmental condition itself.

## Manuscript

Extremophiles are traditionally defined by anthropocentric bias and divided into groups based on environmental conditions (thermophile, psychrophile, piezophile, halophile, acidophile, alkaliphile) (Merino et al. 2019, Schmid et al. 2020). While this condition-based language is descriptive, it can camouflage the molecular-level mechanisms underpinning cellular survival that are always reflective of the environmental conditions. As MacElroy noted in the first published use of the term ‘extremophile’ from the Proceedings of the First European Workshop on Microbial Adaptations to Extreme Environments, “The primary reason for suggesting this artificial classification is ease in verbal and written communication concerning them. It is obvious that any such group cannot be considered exclusive or temporally stable” (MacElroy 1974). Even so, the artificial separation by distinct condition, however, can be misleading. An example is the term ‘polyextremophile’, which came into use to describe extremophiles that cross condition-based groupings. The use of this distinct term suggests that polyextremophiles are rare, in contradiction to field-based observations of extreme environments that are inherent mixes of different physicochemical stressors. In addition, while environmental conditions drive evolution via natural selection, resistance to physicochemical stressors is not intrinsically linked to specific environmental condition, but rather to chemical damages of key cellular components. Such a distinction is missed when using condition-based language rather than chemistry-based descriptors.

The study of life in extreme environments is inherently interdisciplinary, involving chemistry, physics, geology, and biology. This requires both a commonly understood set of terminology between disciplines and a recognition of the unstated, underlaying concepts of each discipline. One such principle in extremophile microbiology is that biological survival is underpinned by biomolecular mechanisms that protect against or repair stress-induced macromolecular damages. This can be clarified by intentionally referring to the chemical changes of cellular components (e.g.: DNA denaturation, loss of membrane stability) rather than employing the traditional, context-specific shorthand of extreme conditions. Such a shift emphasizes a mechanistic rather than descriptive point of view. This enables recognition of generalized patterns concerning biological survival based on macromolecular properties that are lost in a condition-specific approach.

Investigating whether or not there are strict limits to life, what those limits are, and why such limits exist requires a focal switch away from conditions and towards the chemistry of the system. Analyzing the chemical reactions, structures, and molecular mechanisms that enable the viability of micro-organisms provides a more universal basis for evaluating present and past life. It also provides a theoretical framework to extend to novel types of micro-organisms yet to be discovered. Therefore, a deeper examination of the limits of molecular and cellular structures exposed to different physicochemical stressors are necessary.

The basic building blocks of cellular life as we know it are lipids, proteins with their composite amino acids, and nucleic acids (DNA, RNA), all in a liquid solvent (e.g.: water) during active phases of cell metabolism and division. Such (macro-)molecular cellular structures can vary according to the environmental physicochemical parameters, whether occurring in a punctual manner or persisting over the lifespan of the organism, as well as traces of past environments.

Historically, molecular studies of extremophiles developed in tandem with new techniques. Starting in the 1970s, molecular-based approaches produced details on the adaptation and acclimation or acclimatization (laboratory or natural environment, respectively) of each class of biomolecule. These studies showed how biomolecular stability and function were maintained under extreme conditions. Structural studies of individual and small groups of targeted molecules were later combined with -omics, isotopy-based, and other approaches to decipher the molecular mechanisms of damage prevention, response, and repair. These were in turn coupled with advances in cellular and molecular imagery for structural analyses, as well as phylogenetic approaches to determine evolutionary history in micro-organisms. Current methods, including cryo-electron microscopy, provide even more details into changes in cellular and molecular structures. While these data were gathered to study the effects of only one or a small sub-set of extreme physicochemical stressors, changing the point of view towards the cell itself provides a new perspective.

**Figure 1:**
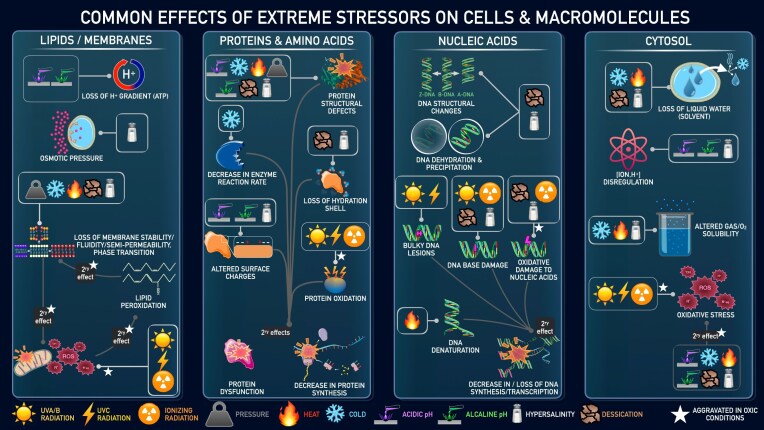
Effects of physicochemical stressors on the main categories of cellular macromolecules (see Table 1 for further details; figure uses images from Servier Medical Art). Multiple stressors can induce the same primary effect, therefore adaptations in response to a natural selection pressure for one stressor may render the same organism resistant to all stressors producing the same type of damage. White stars indicate chemical processes that are aggravated in the presence of oxygen due to altered system chemistry, meaning that in anoxic environments such effects are reduced or negligible.

Drawing on all these sources, Fig. 1 graphically presents a summary of the effects of environmental stressors on cellular components (lipids, amino acids and proteins, nucleic acids, and cytosol). Further details and pertinent references are presented in Table 1. The focus on cellular components reveals both the chemical and structural changes induced by environmental stressors, and how different stressors can produce the same macromolecular changes. From this complex network of information, four key generalities can be drawn:

1) **Microbial survival after exposure to environmental stressors is challenged by damage incurred at the molecular level**.

**Table 1:** Macromolecular damage induced by physicochemical stressors. Common primary damage is indicated in bold, with key common down-stream damage underlined.

Physiochemical stressor	Potential damage to cellular (macro-)molecules (*denotes damage aggravated by the presence of oxygen)				Selected review articles (*non-review articles)
	Lipids/membranes	Proteins/amino acids	Nucleic acids (DNA, RNA)	Cytosol	
**Heat (>60 °C)**	**Membrane lipids excessively mobile, membrane phase transition** (leading to loss of membrane selective-/semi-permeability as fluidity/permeability is too high, disruption to cell bioenergetics)	**Protein denaturation/misfolding** (leading to protein aggregation)	**DNA unwinding, DNA strand denaturation** (leading to exposed single-stranded DNA), loss of DNA replication/transcription/repair (due to denaturation of necessary protein enzymes)	**Dehydration** due to evaporation, **loss of substrate transport** (leading to loss of intracellular biochemistry, arrest of growth and cell division processes), **reduced gas/oxygen solubility**, oxidative stress* in oxic environments (from loss of enzymatic ROS scavengers due to protein inactivation)	Daniel et al. 1996, Daniel and Cowan 2000, Jebbar et al. 2015, Carré et al. 2022
**Cold (<15 °C)**	**Membrane excess structural rigidity, membrane phase transition** (leading to loss of membrane selective-/semi-permeability as fluidity/permeability is too low, disruption to cell bioenergetics)	**Increased structural rigidity** (leading to protein dysfunction), **decrease in enzyme reaction rates** (leads to decreased metabolism, cell cycle slowing/arrest)	Decreased DNA replication/transcription/repair (due to inactivation of necessary protein enzymes)	**Increased oxygen solubility** at low temperature (leading to transient oxidative stress); if cytosol freezing occurs: **dehydration, loss of substrate transport** (leading to loss of intracellular biochemistry, arrest of growth and cell division processes)	Maayer et al. 2014
**Acidic pH**	**Loss of controlled H+ gradient for ATP production** (proton motive force too high due to excessive extracellular proton concentrations, leading to disruption to cell bioenergetics)	**Protonation resulting in changes in surface amino acid residue charges** depending on their pKa (leading to chemical bond disruptions, structural instability/protein denaturation/misfolding, decrease in protein synthesis due to decrease in ribosome activity, loss of enzymatic ROS scavengers, protein Fe-S prosthetic group destabilization)	Decreased DNA replication/transcription/repair (due to protonation and subsequent inactivation of necessary protein enzymes)	**Accumulation of intracellular H** ^+^, oxidative stress* in oxic environments (due to cytosolic and membrane protein structural destabilization and loss of function leading to increased ROS production including from electron transport chain destabilization, protein Fe-S prosthetic group destabilization and increased iron solubility)	Baker-Austin and Dopson 2007, Krulwich et al. 2011
**Alkaline pH**	**Loss of controlled H+ gradient for ATP production** (proton motive force too low due to insufficient extracellular proton concentrations, leading to disruption to cell bioenergetics)	**Deprotonation resulting in changes in surface amino acid residue charges** depending on their pKa (leading to chemical bond disruptions, structural instability/protein denaturation/misfolding, decrease in protein synthesis due to decrease in ribosome activity, loss of enzymatic ROS scavengers, protein Fe-S prosthetic group destabilization)	Decreased DNA replication/transcription/repair (due to deprotonation and subsequent inactivation of necessary protein enzymes)	**Loss of intracellular H^+^**, indirect evidence for oxidative stress* in oxic environments (hypothesized to be due to elevated yields of ROS resulting from Cys deprotonation forming reactive thiolate, electron transport chain destabilization, and loss of enzymatic ROS scavengers due to protein inactivation from deprotonation), disruption in osmoregulation (due to membrane protein transporter dysfunction or disequilibrium for monovalent cation/proton antiporters)	Padan et al. 2005, Krulwich et al. 2011
**Desiccation**	**Loss of hydration of membrane lipids and proteins** (leading to electron transport chain destabilization *in oxic environments this results in lipid peroxidation, membrane phase transition due to increased van der Waals interactions between lipids	**Protein dehydration/lack of solvation, disulphide bridge formation, protein-DNA crosslinking, Maillard reaction** between Lys/Met and reducing sugars (leading to protein denaturation/misfolding, protein aggregation), low levels of protein oxidation in anoxic environments from redox reactions with transition metals	**DNA structural changes** (conversion of DNA structure from B-form to A-form (short, wide helix), **DNA dehydration leading to aggregation/precipitation** (known as “salting out”), oxidative damage* to nucleic acids, **mechanical stress from cell shrinkage and depurination/depyrimidination/base loss** (leading to DNA strand break accumulation), loss of DNA replication/transcription/repair (due to inactivation of necessary protein enzymes) leading to cell cycle arrest	**Dehydration** (leading to low water activity with increased solute concentrations and pH alteration, macromolecular crowding, lack of solvent for intracellular transport, cell shrinkage, loss of intracellular biochemistry, arrest of growth and cell division processes), oxidative stress* in oxic environments (elevated yields of ROS resulting from electron transport chain destabilization, and loss of enzymatic ROS scavengers due to protein inactivation)	Wang et al. 1979 *, Cox 1993, Potts 1994, Billi and Potts 2002, França et al. 2007, Grzyb and Skłodowska 2022, Billi and Potts 2002, Cox 1992, Potts 1994
**Hyper salinity**	**Osmotic pressure** due to high extracellular ion concentrations, **loss of mobility** and **dehydration of phosphate head groups** due to formation of salt bridges between negatively charged lipids and environmental cations (leading to increased van der Waals interactions between lipids and membrane phase transition), **loss of hydration of membrane proteins** (leading to electron transport chain destabilization and *in oxic environments this results in lipid peroxidation)	**Protein dehydration/lack of solvation** due to low water activity, **protein charge screening, chaotropicity** (i.e.: increased macromolecular instability) or **kosmotropicity** (i.e.: increased macromolecular rigidity) depending on the brine composition, leading to protein aggregation and precipitation known as **“**salting out”), protein dysfunction, macromolecular aggregation)	**DNA structural changes** (conversion of DNA structure from B-form (right-handed helix turn) to Z-form (left-handed helix turn, unstable), **charge screening** leading to increased macromolecular stability of the DNA helix, **chaotropicity** (i.e.: increased macromolecular instability) or **kosmotropicity** (i.e.: increased macromolecular rigidity) depending on the brine composition and the molecule type—RNA is more sensitive than DNA to chaotropicity), oxidative damage to nucleic acids including clustered DNA damage that can lead to the formation of DNA strand breaks during repair processes, loss of DNA replication/transcription/repair (due to inactivation of necessary protein enzymes) leading to cell cycle arrest, **DNA dehydration leading to aggregation/precipitation** (known as “salting out”)	**Dehydration** due to intracellular water following the osmotic gradient towards the cell exterior (leading to low water activity with increased solute concentrations and pH alteration, macromolecular crowding, lack of solvent for intracellular transport, cell shrinkage), **low oxygen/gas solubility** in saturated brines (lower ATP production via anaerobic metabolisms), oxidative stress* in oxic environments (elevated yields of ROS resulting from electron transport chain destabilization, and loss of enzymatic ROS scavengers due to protein inactivation)	Sherwood et al. 1991 *, Misra and Honig 1996 *, Zaccai 2004, Hallsworth et al. 2007 *, Kish and DiRuggiero 2012, Kellermann et al. 2016, Lee et al. 2018, Kriegler et al. 2021 *, Carré et al. 2022
**High (hydrostatic) pressure**	**Membrane phase transition, loss of membrane selective-/semi-permeability** (i.e.: fluidity/permeability too low, leading to disruption to cell bioenergetics)	**Mechanical stress and resulting loss of structural stability** (leading to protein dysfunction, decrease in protein synthesis due to decrease in ribosome activity)	Decreased DNA replication/transcription/repair (due to inactivation of necessary protein enzymes) leading to cell cycle arrest	Indirect evidence for oxidative stress	Aertsen et al. 2004 *, 2005 *, Oger and Jebbar 2010, Jebbar et al. 2015, Carré et al. 2022
**High solar radiation (UVA, low UVB))**	**Electron transport chain destabilization** (leading to elevated yields of ROS depending on oxygen availability, disruption to cell bioenergetics) *in oxic environments this results in lipid peroxidation leading to loss of membrane function and stability/fluidity (particularly for UVB)	*UVB an to a lesser extent UVA:* **Direct and indirect damage to amino acids** (aromatic functional groups of Trp, Tyr, Phe, His along with S-containing Met, Cys, and disulfides) (leading to peptide fragmentation, macromolecular aggregation and protein dysfunction, damage to photosynthetic pigments leading to disruption to cell bioenergeticsin photosynthetic organisms)	*UVA:* **Mainly indirect effects (photosensitization reactions leading to DNA base oxidation**, clustered DNA damage can lead to the formation of DNA strand breaks during repair processes), limited formation bulky DNA lesions blocking DNA replication/transcription (cyclobutane pyrimidine dimers, Dewar valence isomers) leading to cell cycle arrest; *UVB:***some direct UV absorption by DNA causing damage to pyrimidine bases** forming bulky DNA lesions blocking DNA replication/transcription (dimerization of adjacent pyrimidine bases, cyclobutane pyrimidine dimers, 6–4 photoproducts) leading to cell cycle arrest, **indirect effects (photosensitization** reactions leading to DNA base oxidation, clustered DNA damage can lead to the formation of DNA strand breaks during repair processes)	**Free radical production** via Type I (e^-^, H_2_ transfer) or Type II (energy transfer to O_2_) mechanisms involving sensitizer molecules) (leading to oxidative stress* in oxic environments)	Cadet et al. 2005, Pattison and Davies 2006, Pattison et al. 2011, Santos et al. 2013 *, Schuch et al. 2013, Bacellar and Baptista 2019, Amador-Castro et al. 2020
**UVC radiation**	**Electron transport chain destabilization** (leading to disruption to cell bioenergetics, elevated yields of ROS *in oxic environments this results in lipid peroxidation) leading to loss of membrane function and stability/fluidity	**Direct and indirect damage to amino acids** (aromatic functional groups of Trp, Tyr, Phe, His along with S-containing Met, Cys, and disulfides) (leading to peptide fragmentation, macromolecular aggregation and protein dysfunction, damage to photosynthetic pigments leading to disruption to cell bioenergetics in photosynthetic organisms)	**Direct absorption by DNA** (maximal at 254 nm) (leading to formation of bulky DNA lesions in the form of 6–4 photoproducts blocking DNA replication/transcription processes leading to cell cycle arrest)	**Free radical production** via Type I (e^-^, H_2_ transfer) or Type II (energy transfer to O_2_) mechanisms involving sensitizer molecules) (leading to oxidative stress* in oxic environments)	Chatterjee and Agarwal 1983 *, Douki and Cadet 2003, Pattison and Davies 2006, Pattison et al. 2011, Santos et al. 2013 *
**Ionizing radiation**	**Lipid peroxidation** from water radiolysis (leading to loss of membrane function and stability/fluidity)	**Protein oxidation** (leading to loss of protein stability/function resulting in loss of key cellular processes including DNA repair and metabolism)	**Oxidative damage to nucleic acids** (clustered DNA damage can lead to the formation of DNA strand breaks during repair processes), loss of DNA replication/transcription/repair (due to inactivation of necessary protein enzymes)	**Radiolysis of cytosolic water** producing ROS (*higher ROS yields in oxic environments) (leading to oxidative stress)	Chatterjee and Agarwal 1983 *, Cadet et al. 1999, 2003, 2005, Daly 2009

As summarized in Fig. 1 and detailed in Table 1, key biomolecules are chemically modified by physicochemical stressors. This affects the resulting macromolecular structures and cellular functions in a ripple of effects. Not only does this perspective clarify the cause and effect of environmental stressors, but it also helps more accurately characterize the stresses faced by micro-organisms in extreme environments. For example, micro-organisms in high salt environments are more precisely challenged by ‘salinity stress’. This generalized term is inclusive of low water activity, high osmotic pressure, chaotropicity, or kosmotropicity [due to ion types, and ionic stress (due to ion concentrations]. Additionally, saturated brine environments tend to experience periodic desiccation and high solar irradiation (UVA, UVB), resulting in further potential damage to cellular macromolecules (see Fig. 1 and Table 1). High hydrostatic pressure includes mechanical stresses to membranes and protein complexes affecting protein stability, and indirect evidence for oxidative stress (Aertsen et al. 2004, 2005, Oger and Jebbar 2010, Jebbar et al. 2015, Carré et al. 2022) damaging cellular components. These macromolecular alterations in turn lead to the loss of translational activity, cell mobility, membrane semi-permeability, bioenergetics at the cellular level (Aertsen et al. 2004, 2005, Oger and Jebbar 2010, Jebbar et al. 2015, Carré et al. 2022). Therefore, the effects of stressors on cellular (macro)molecules must be examined when evaluating the limits of life.

2) **Damage-susceptibility of cellular (macro)molecules varies by structure, chemical composition, and surrounding molecules**

Extremophile micro-organisms differ from mesophiles based on adaptions of cellular structures and key biomolecules to protect against and/or repair damages induced by physicochemical stressors. These adaptations have been extensively reviewed elsewhere (Daly 2009, Oger and Jebbar 2010, Jebbar et al. 2015, Orellana et al. 2018, Amador-Castro et al. 2020, Schmid et al. 2020, Carré et al. 2022). From studies of the damage to cells and isolated molecules, to single and multiple stressors, some patterns have begun to emerge relevant to the limits of life and detection of biogenic molecules in extreme environments. Although cell surface structures interface with the surrounding environment, cell wall thickness does not necessarily confer added resistance. Cell walls can be thin and still resistant to environmental extremes, as in the case of S-layers in extremophilic archaea from thermoacidophiles to halophiles (Albers and Meyer 2011). Inversely, cell walls can be thick multi-layered structures and relatively sensitive to mechanical and chemical damage as in the case of mesophilic bacteria (Albers and Meyer 2011). Membrane lipids with ether linkages are less susceptible to damage from heat, pH and oxidation than those with ester linkages (Choquet et al. 1994, Driessen and Albers 2007). RNA is more susceptible to damage than DNA, and single-stranded DNA is more fragile compared to double-strand helices. Guanine bases are hot-spots for ionizing radiation damage whereas pyrimidines are more susceptible to UV-induced damage (Cadet et al. 2003, 2005). Protein Fe-S clusters are highly sensitive to oxidative stress via Fenton chemistry, particularly in the presence of oxygen (Daly 2009). Amino acid functional groups have varying responses to pH based on their pKa, and to UV radiation based on their structure (aromatic groups are more UV-sensitive) (Pattison et al. 2011).

Once damaged, not all biomolecules have equal capacity for repair. Damage to DNA can potentially be repaired, if repair proteins remain functional. However, damaged proteins must be degraded and replaced by biosynthetic pathways rather than repaired. Thus, management of protein damage is likely more significant than even DNA damage for cellular viability. For this reason, since the early 2000s the study of radioresistance mechanisms has refocused on protein oxidation rather than DNA damage (Daly 2009).

Prevention of damages to proteins and other key biomolecules in extremophiles range from enzymatic processes (e.g: antioxidants) to molecular chaperones (e.g.: heat/cold shock proteins) to diverse passive mechanisms involving small metabolites (< ∼1 kDa). For instance, sugars such as trehalose, along with free amino acids and their derivatives such as ectoine can accumulate within the cytosol. These compatible solutes have been shown to help maintain intracellular water and counteract osmotic, oxidative, and mechanical stresses under high and low temperatures (Santos and Costa 2002, Czech et al. 2018), high hydrostatic pressure (Iwahashi et al. 1997, Czech et al. 2018), osmotic stress (Roberts 2005), ionizing radiation exposure (Webb and DiRuggiero 2012, Czech et al. 2018), and desiccation (Potts 1994, Czech et al. 2018). Mycosporine-like amino acids can protect membrane photosystems from UV damage (Amador-Castro et al. 2020). Accumulation of cytosolic Mn^2+^ complexes (Daly et al. 2004, Webb et al. 2013), halides (Kish A et al. 2009), and α-keto acids such as pyruvate (Kim et al. 2016) provide protection from oxidative damage resulting from a range of different environmental stressors. Lipid pigments such as carotenoids and melanin are found in all three domains of life (Bacteria, Archaea, and Eukarya), and offer UV protection as free radical scavengers and physical attenuators (Cockell and Knowland 1999, Farci et al. 2016, Grivard et al. 2022, Koch et al. 2023, Xie et al. 2024). Furthermore, negatively charged intracellular polyphosphate granules can sequester positively charged toxic heavy metals and/or radionuclides (Suzuki and Banfield 2004).

3) **Local physicochemical states alter the chemistry underpinning microbial survival**.

**Figure 2:**
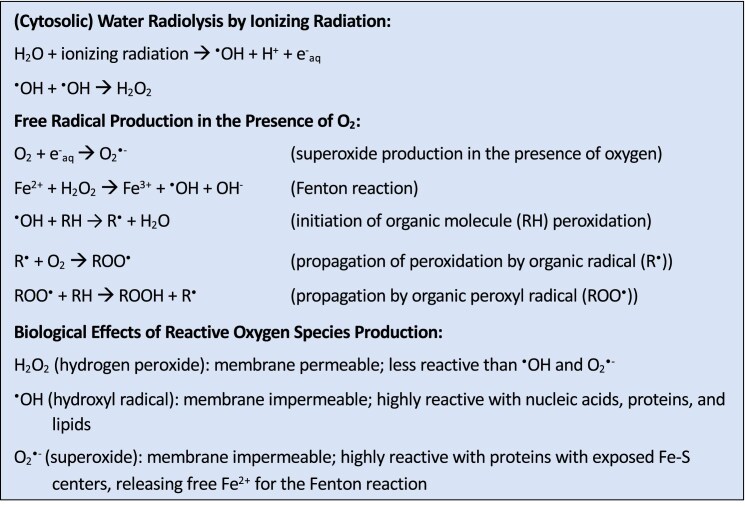
Intracellular chemistry of free radical production from ionizing radiation (modified from Daly 2009, Bobrowski 2017 and Zielinski and Pratt 2017).

Differences in atmosphere, in biogeochemical interactions, in energy sources, and in hydration state can all alter cellular chemistry thereby changing potential cytotoxicity. In the case of exposure to ionizing radiation, the presence of oxygen increases production of both highly reactive hydroxyl radicals through Fenton chemistry and superoxide radicals (see Figs. 1 and 2). This in turn leads to inactivation of essential enzymes for DNA repair and metabolic functions (Daly 2009, Bobrowski 2017). Oxygen-preferring micro-organisms (including facultative anaerobes) must therefore balance higher energy production yields compared to anoxygenic ATP formation reactions, with potential toxicity from reactive oxygen species (ROS) production. A mechanism common to aerobic, radioresistant prokaryotes is limiting oxidative damage by increased intracellular Mn/Fe ratios. Replacement of intracellular Fe, with Mn both restricts the Fenton reaction and increases scavenging of ROS by Mn-peptide complexes (Daly 2009, Daly et al. 2010, Culotta and Daly 2013). These non-enzymatic mechanisms are in addition to the actions of free radical scavenging enzymes, DNA repair systems, and protein turn-over. However, in anoxic environments the damage to nucleic acids, proteins, and lipids incurred by reactions with hydroxyl radicals from water radiolysis would not exacerbate damage through extensive secondary reactions with O_2_ forming superoxide. Reduced ROS production is significant for life in anoxic environments such as older (>1 Ma) deep subseafloor sediments. In such sediments, the advantage of lower ROS is cumulative with the radiolytic production of H_2_ (see Fig. 2) that serve as the primary electron donor for microbial metabolisms (Sauvage et al. 2021).

Geo-biological interactions can also increase microbial resistance to UVC via shielding minerals such as halite (Cockell and Raven 2004, Fendrihan et al. 2009), occurring for halophiles that are entrapped inside salts during evaporation. Ferric iron (Fe^3+^) containing minerals also have UV-attenuating capacity (Godin et al. 2020). However, whether the effect is protective or degradative for lipids, proteins, and amino acids depends on the salt (Gault and Cockell 2021, Kriegler et al. 2021, Bourmancé et al. 2025) and mineral compositions (Laurent et al. 2019). Mineralogical protection of microbial cells reinforces the close links between geology, biology, and chemistry at the limits of life.

Availability of energy sources such as in the deep subsurface presents a potential metabolic limitation for microbial life. The ultra-slow growing extremophilic ‘aeonophiles’ (Lloyd and Steen 2025) adapt through reduced rates of metabolic activity and cell division over geologically-relevant time scales. Traditional methodologies for measuring metabolic activity (e.g.: *in situ* isotopy) present technical challenges in low energy environments on Earth due to limited accessibility, low biomass, and physicochemical parameters limiting equipment functioning (Lever et al. 2015, Lloyd 2021). Additionally, extremely low rates of metabolic activity require timescales for *in situ* detection beyond what is practically feasible. Targeted metabolic approaches will miss currently unknown types of metabolisms. Standard *in situ* methodologies often cannot distinguish dead cells from dormant or inactive cells as a result of misfunctioning reagents in extreme environments or incompatibility with the unique physiology of extremophiles. Nevertheless, studies have shown that such ‘aeonophiles’ favor recycling of membrane lipids over *de novo* biosynthesis as an energy-saving mechanism in both the marine deep subsurface (Takano et al. 2010) and hypersaline sediments (Thomas et al. 2019). Recycling produces unique lipid signatures such as wax ester storage lipids (Thomas et al. 2019).

Temporary loss of internal water inducing a dormancy state is a survival strategy in unfavorable environments for a wide range of organisms, from spores of *Bacillus* and fungi to the contracted tardigrade tun state. Hydration state is a key parameter used to distinguish between dormant and active physiological states. Even so-called ‘xerophiles’ that are defined by desiccation resistance, need liquid water at some point in their life cycle as a solvent for essential cytosolic biochemical reactions (see Fig. 1). Therefore, water activity, the vapor pressure of a solution compared to that of pure water, is one of the key delimiters of life. This applies both to known life on Earth (Stevenson et al. 2015) as well as to theoretical evaluations of the potential for microbial life on other planetary bodies such as Venus (Hallsworth et al. 2021) and Mars (Tosca et al. 2008, Fox-Powell et al. 2016). Extremophiles can survive at lower water activities through adaptations of their cytosols. Accumulations of inorganic salt ions such as K^+^ (Engel and Catchpole 2005, Lee et al. 2018), organic metabolites such as glycerol or hygroscopic osmolytes such as trehalose (Williams and Hallsworth 2009, Stevenson et al. 2014, 2015) are all strategies employed by extremophiles in low water activity environments. The exopolysaccharide matrix of biofilms also provides localized protection to cells within, acting as cryoprotectants in cold environments and increasing adherence for metal ion sequestration from mineral surfaces in metal-rich, acidic environments (Bhat and Roach 2025).

Lack of liquid water is of particular interest in astrobiology for planets such as Mars that no longer have stable liquid water at their surface. Determining the potential for microbial life on Mars involves controlled experiments combining the radiation environment with desiccation (Chander et al. 2026). Space exposure experiments of radiation resistance in micro-organism therefor capitalize on dehydration-resistant species. Samples are prepared as stable, non-motile samples in thin layers (ideally monolayers) for exposure to the space environment (Olsson-Francis and Cockell 2010, Cottin et al. 2017). Samples have traditionally been rehydrated after return to Earth to test for survival and repair mechanisms. However, data interpretation must account for the fact that the main source of ionizing radiation damage to cells, ROS production from radiolysis of intracellular water, is intentionally absent. This is both for scientific reasons (i.e.: to study the absolute limits of radiation tolerance in terrestrial micro-organisms) and technical reasons as fluidics and *in situ* analytical technology for spaceflight is only just becoming available (Elsaesser et al. 2023). Direct exposure to space radiation of fully hydrated and metabolically active forms of such micro-organisms show markedly reduced survival even after irradiation in anoxic environments, based on ground-based simulations (Beblo-Vranesevic et al. 2017). Conversely, UVC exposure of DNA in a dry state (either as in bacterial spores, or more importantly outside of a cell) leads to the formation of a unique chemical damage type; spore photoproducts (Pattison and Davies 2006). This shows the importance of hydration state to chemical damage of biomolecules.

4) **The same macromolecular chemical and/or structural damages produced by different stressors can be resisted by the same cellular mechanisms**.

Figure 1 shows that different conditions can produce similar macromolecular damages. Radioresistance is the clearest illustration of the difference between resistance phenotypes resulting from selection pressure, and resistances to secondary stressors enabled simply as a consequence of similarities in the induced damage to cell components. Radioresistance has been identified in micro-organisms from such diverse environments as desert soils (Chanal et al. 2006), hypersaline brines (Kottemann et al. 2005), and anoxic deep-sea hydrothermal chimneys (Jolivet et al. 2003). The commonality, therefore, is not the environment or even the sub-category of extremophile, but rather the molecular damage induced for which all these species have mechanisms for preventing and/or repairing. For oxygen-preferring organisms like *Deinococcus radiodurans* and *Halobacterium salinarum*, the commonality is oxidative stress. Both species are exposed to desiccation in their natural habitats, but are nevertheless resistant to UVC radiation, ionizing radiation, and high hydrostatic pressure, whereas *Escherichia coli* is sensitive to all four stressors (Arrage et al. 1993, Mattimore and Battista 1996, Baliga et al. 2004, Daly et al. 2004, Kottemann et al. 2005, Bauermeister et al. 2009, Daly 2012, Kish et al. 2012). This occurs because UVC radiation, ionizing radiation, and high hydrostatic pressure all induce oxidative damage to proteins, DNA, and lipids (Aertsen et al. 2005, Cabiscol et al. 2010) (see Fig. 1 and Table 1). In anoxic environments, however, desiccation tolerance is decoupled from resistance to ionizing and UV radiations (Beblo et al. 2011, Beblo-Vranesevic et al. 2018), thus reflecting different chemistries from those in the presence of oxygen leading to the preservation of cellular macromolecules (Webb and DiRuggiero 2012).

Determining the molecular damage pathways enables an explanation of the survival ability of extremophiles extending beyond what can be explained by natural selection based only on environmental stressors in their native habitat. As shown in Fig. 1 and described in Table 1, many physicochemical stressors produce the same cellular damage, either directly or as a consequence of a chain of events within the cell. Loss of free water in the cell cytosol (dehydration) for example can result from desiccation, evaporative loss from heat, freezing, and extremely high solute content (salts or compatible solutes). Disruption of cell bioenergetics at cell membranes can be caused by extremes in temperature and/or pH, desiccation, hypersalinity, high pressure, and exposure to radiation.

However, the limits of life are delineated not only by physicochemical stressors, but also the specific cellular and molecular adaptations accumulated over the evolutionary history of cellular life. Adaptations to certain stressors can be either synergistic or antagonistic to survival under multiple extremes. The absence of detectable life in specific multi-stress environments has been noted by both metadata analyses (Harrison et al. 2013) and field studies of extreme environments used as analogues for planetary environments. For example, the Dallol geothermal field in Ethiopia is characterized by extreme heat, acidic pH, and hypersalinity. The lack of detectable micro-organisms in the most extreme niches of Dallol were hypothesized to be in part the consequence of incompatible adaptations to extreme acidity and extreme salinity at the membrane level concerning ion transport and bioenergetic processes (Belilla et al. 2019). Accumulations of intracellular K^+^ ions are compatible with both hypersalinity and heat. The chemical stabilizing effects of K^+^ are exploited by extremely halophilic archaea in osmotic adaptation (‘salt-in’ osmoprotective strategy) and stabilization of acidic cystosolic proteins by solvation (rather than hydration) in the face of low water availability. These effects also stabilize the nucleic acids of thermophiles against heat-induced damage (Grosjean and Oshima 2007). However, acidic pH and heat can both destabilize membrane permeability and controlled ion transport for H^+^, K^+^, Na^+^, and Cl^-^ in hypersaline environments potentially leading to disruptions in cell bioenergetics. The interplay between cellular damage induced by physicochemical stressors and adaptations to extreme environments are clearly complex.

### Implications

The use of condition-based categories for extremophiles was originally intended as a descriptive convention. While useful within microbiology, it is limited for interdisciplinary communication as it requires prior knowledge of a set of unstated principles at the cellular and molecular scale. References to the condition can also limit comparisons between the survival mechanisms of extremophiles. The un-intended result is a difficulty in determining generalities concerning the limits of life. It also prevents predictions about new micro-organisms yet to be discovered in regions that are difficult to access.

A focus on the chemical and molecular underpinnings of extremophile survival, rather than conditions, provides interdisciplinary common ground between microbiologists, biochemists, organic (geo-)chemists, and atmospheric scientists. This also provides a more rational basis for defining the limits of life. Applying this logic, it becomes apparent that microbial survival at the organismal level is in fact a by-product of protection against and repair of any damages occurred at the molecular level. Cross-comparison of the resistances of cellular (macro)molecules between different physicochemical parameters enables a decoupling of the concept of polyextremophily from the local conditions of the natural habitat. Extreme environments are by nature of their planetary context (geochemical, hydrological, and atmospheric) combinations of physicochemical extremes. Therefore, it is logical that micro-organisms inhabiting such environments are adapted to thrive under multiple descriptive categories of conditions. However, survival of extremophiles outside of these natural selection pressures can only be understood through the lens of cellular and molecular chemistry. Taken together, these factors explain why micro-organisms can resist extreme conditions foreign to their native habitats, as exemplified by the radioresistance of *D. radiodurans* despite it not being native to radioactive conditions.

This perspective has a direct implication for understanding the limits of life. It allows both a mechanistic understanding of known systems, and a predictive framework for novel biological systems yet to be studied based on established (bio-)chemical principles. For example, no micro-organism identified to date is able to directly use ionizing radiation as an energy source, only the byproducts of radiolytic and redox reactions (Lin et al. 2006, Sauvage et al. 2021), but perhaps a novel energy harvesting system could theoretically perform this function for an organism following a distinct evolutionary pathway.

A more chemical-based approach is also more conducive to investigations of traces of ancient micro-organisms. Applications include paleoenvironment reconstruction from microbial metabolites and lipids, as well as studies of microbial fossilization and diagenesis processes. It is also useful for studies of the slow-growing aeonophiles and long-preserved dormant microbial forms.

A focus on specific chemistries aids experimental design. It contributes to successful down-selection of molecules that can be considered as potentially microbial in origin, as well as how to extend this definition to potential novel biochemistries following standard chemical principles accounting for the effects of different physicochemical parameters. It also provides a more precise basis from which to optimize novel automated extraction protocols for biomolecules from natural samples. The appropriate selection of specific standards for biomolecule detection instrumentation is best guided by the types of chemical structures and modifications expected for a given combination of physicochemical parameters. Even the choice of an extremo-philic (absolute requirement of ‘extreme’ conditions for growth and cell division), extremo-tolerant (passive resistance to ‘extreme’ conditions), or extremo-resistant (possessing molecular protection and repair mechanisms) model organisms depends on if the scientific question concerns the absolute limits of life or resistance to changing physicochemical parameters. These choices are more limited with a focus only on the relevant condition, rather than on the potential cellular chemistries that could result in unexpected survival.

While life is only known to exist on Earth, the concepts of the limits of life and habitability are also studied in reference to the potential for life on other planets in our solar system as well as exoplanets. Extremophiles provide practical models needed to study the limits of life. Astrobiology in turn provides the perspective to examine the fundamental questions including why such limits exist, and if there are even absolute limits to a form of hypothetical cellular life based on alternative planetary contexts. Such studies expand the interdisciplinary pool of relevant expertise to include astronomy, planetary science, and planetary protection under the general umbrella of ‘astrobiology’.

For astrobiology, a focus on cell chemistry provides a baseline for selection of appropriate analogue molecules and organisms for the development of theoretical model and novel detection methodologies. A focus on the chemical limits of cellular macromolecules also provides parameters for site selection of astrobiology analog studies and planetary exploration missions based on the cell itself, rather than the environmental conditions. A chemistry-focused approach also provides a more concrete basis for describing habitability in the universe including distant exoplanets (e.g.: Habitable Worlds Observatory, Large Interferometer For Exoplanets). The data from such missions must in turn be interpreted following rigorous guidelines for potential detection of past or present biology, taking into consideration evidence from molecular, structural, cellular, ecosystem, and planetary levels (Neveu et al. 2018, Barge et al. 2022, Meadows et al. 2022, Malaterre et al. 2023).

Environmental condition-based points of view are limited to studying the effects of a specific set of environmental parameters on microbial life. Using a chemistry-based perspective enables a broader analysis of the fundamental limits of life on a mechanistic level. A clear understanding of the chemical and structural limits of lipids, amino acids, nucleic acids, and the cytosolic solvent is required to determine if there are definitive limits to life, and if so, what those limitations are. From this framework, comparisons can be made across different conditions to provide general principles. This perspective is also more conducive to interdisciplinary work inclusive of geology, physics, chemistry, and biology at different scales. Therefore, while descriptive terms are useful in science, it is important for discussions of extremophiles to focus on the concept that life resists chemistry, not conditions.
